# Utilisation of cardiac pacemakers over a 20-year period: Results from a nationwide pacemaker registry

**DOI:** 10.1007/s12471-016-0880-0

**Published:** 2016-08-25

**Authors:** L. M. de Vries, W. A. Dijk, C. A. M. Hooijschuur, M. J. G. Leening, B. H. C. Stricker, N. M. van Hemel

**Affiliations:** 1 Department of Epidemiology, Erasmus MC – University Medical Center Rotterdam, Rotterdam, The Netherlands; 2 Thorax Center, University Medical Center Groningen, Groningen, The Netherlands; 3 Department of Cardiology, Erasmus MC – University Medical Center Rotterdam, Rotterdam, The Netherlands; 4 Department of Epidemiology, Harvard T.H. Chan School of Public Health, Boston, MA USA; 5 Department of Cardiology, University Medical Center Utrecht, Utrecht, The Netherlands

**Keywords:** Pacemaker, Leads, Utilisation, Indication, Type, Registry

## Abstract

The implantation of cardiac pacemakers has become a well-established therapy for conduction disorders and sinus node dysfunction. In many countries pacemaker registries have been initiated in order to collect information on patient characteristics, trends in numbers and the types of pacemakers used, to identify problematic devices, and for safety monitoring. For this utilisation study the Central Pacemaker Patients Registration (CPPR) from the Netherlands Pacemaker Registry Foundation (CPPR-SPRN) containing data collected for more than 20 years was used. During this period nearly 97,000 first pacemakers were implanted. Analyses show an increase in the rate of implanted devices. The change in pacemaker type from VVI to DDD, followed by biventricular stimulation, is reflected by the number of simultaneously implanted leads, which is partly a consequence of cardiac resynchronisation therapy. Our data demonstrate that indications for implantation and type of pacemaker are comparable with other European countries.

## Introduction

Advantages of cardiac pacing have been established in the past decades for conventional indications such as conduction disorders, and with new applications for treatment of arrhythmias and heart failure being added, clinical investigation is ongoing. Implantation of cardiac pacemakers has prolonged the lives and improved the quality of life of many patients [1–3]. Despite these advantages, implantation of devices is also inevitably associated with complications and may be prone to product defects. This was illustrated by several major cases and recalls in the past, such as the Accufix leads for cardiac pacemakers [4–7].

In many countries device registries were initiated by individual cardiologists or national societies in order to gain insight into patient characteristics, trends in numbers and the types of pacemakers used, to inform participating centres about problematic devices, and to exchange information between countries [8–10]. However, most of these registries were restricted to a limited number of hospitals or geographical areas. In the Netherlands, registration with the intention to record every pacemaker implanted in Dutch hospitals was initiated in 1982. This registry, maintained by the Netherlands Pacemaker Registry Foundation (SPRN), was kept until 2008 after which the Netherlands Society of Cardiology started a new registration: the Dutch ICD and Pacemaker Registration (DIPR), which was recently integrated into the overarching National Cardiovascular Data Registry (NCDR).

The objective of the current analysis was to study changes in the utilisation of cardiac pacemakers for new implantations over a period of more than 20 years in a country with the nearly nationwide pacemaker registry CPPR-SPRN.

## Methods

### Setting

Data were retrieved from the Central Pacemaker Patients Registration (CPPR) from the Netherlands Pacemaker Registry Foundation (CPPR-SPRN). In 1982, CPPR-SPRN was established and the computerised Central Pacemaker Patients Registration was started. The aims of the registry were to get an overview of: 1) patient and implant characteristics; 2) trends in types of pacemakers and leads; and 3) the annual number of implants per clinic and nationwide. Furthermore, the objectives of the registry were to inform the participating clinics and recipients about quality issues with pacemakers and leads, to exchange information with other European countries, and to increase indirect patient care by furnishing information to clinics about pacemakers implanted elsewhere and to patients about pacemaker centres in other countries.

Cardiologists and pacemaker technicians were requested to register data on the patient, device and leads on the pacemaker card. Each recipient of a pacemaker was registered in the database. Data on symptoms, indication and diagnosis, brand of pacemaker and leads, type, follow-up visits, explantation, hospital transfer and death were registered according to European Registry Guidelines established in 1982 and later. When CPPR-SPRN ended its registration activities in December 2007, data on 174,405 first implantations and replacements, with 204,920 leads, had been recorded for 136,342 patients during 25 years [8].

### Monitoring and validation of data

Until 1989, data were centrally registered by sending a carbon copy of the pacemaker card to the registry. From 1989 onwards, digitalised registration was used with automatic communication between the central registration computer and the local computer of the implanting centre. During the daily conversions into the database, multiple checks were performed on: missing data, conformation with already stored information and plausibility [11]. Additionally, the data were periodically returned to the clinics for correction purposes. A validation process in order to obtain better insight into the quality of the database was performed in 1997 [12].

### Cohort

Based on the available data, an inception cohort of patients was formed containing all first implanted pacemakers during the period 1 January 1984 until 1 January 2006. A total of 353 implantations were excluded because of inconsistencies in the registered data, such as a new implantation being registered after the supposed date of death of a patient, or the same pacemaker registered more than once with different explantation dates, leaving 96,900 first implantations for analysis.

At the start of the registration in 1984, 120 hospitals participated, some of which were sub locations of the same hospital but participated independently. During the study period several locations and/or hospitals merged and the registry was continued under one account, leaving 101 participants in the registry. General population data were obtained from Statistics Netherlands (CBS, www.cbs.nl/en-GB/).

### Analysis

We computed straightforward descriptive statistics for the aetiology, pacemaker types, and the prevalences of symptoms and ECG characteristics as percentages of all first implantations. For implantation rates, we calculated Poisson intervals. For the comparison of normally distributed continuous determinants, we used independent samples T‑tests while categorical determinants were compared with chi-square statistics. All statistical analyses were performed in IBM SPSS Statistics version 20.0 (IBM Corp., Somers, New York, USA).

## Results

Between 1 January 1984 and 1 January 2006, 96,900 first pacemakers were implanted (Table 1). This corresponds to an average number of implantations of more than 4600 devices per year, varying from a total of 3236 first implantations in 1984 (225 implants per million inhabitants) to 6901 first implantations in 2005 (423 implants per million inhabitants).

**Table 1:** Baseline characteristics of the patients at implantation of first pacemaker

	Women^a^	Men^a^	*p*-value for difference^b^	All first implantations
Total number of first implantations	45,661 (47,1)	51,164 (52.8)	<0.001^c^	96,900
Mean age, years (SD)	74.6 (12.6)	71.4 (13.2)	<0.001	72.9 (13.0)
Median age, years	77.0	74.0	–	75.0
Age ≥60 years, *n* (%)	41,457 (90.8)	43,637 (85.3)	<0.001	85,165 (87.9)
Age ≥80 years, *n* (%)	17,489 (38.3)	12,208 (23.9)	<0.001	31,569 (32.6)
ECG, *n* (%):				
Sick sinus syndrome	20,166 (44.2)	20,847 (40.7)	<0.001	41,026 (42.3)
Heart block	17,612 (38.6)	20,050 (39.2)	0.049	37,682 (38.9)
Bundle branch block	1537 (3.4)	2582 (5.0)	<0.001	4121 (4.3)
Normal sinus rhythm (with or without abnormal EPS^d^) or not documented	940 (2.1)	1164 (2.3)	0.021	2104 (2.2)
Other	541 (1.2)	691 (1.4)	0.022	1232 (1.3)
Unknown/uncoded/unspecified	4865 (10.7)	5830 (11.4)	<0.001	10,735 (11.1)
Symptoms, *n* (%):				
Syncope	13,004 (28.5)	14,651 (28.6)	0.592	27,672 (28.6)
Dizzy spells	12,749 (27.9)	12,973 (25.4)	<0.001	25,728 (26.6)
Bradycardia	9,485 (20.8)	10,908 (21.3)	0.037	20,398 (21.1)
Dyspnoea/heart failure	2396 (5.2)	3064 (6.0)	<0.001	5462 (5.6)
None/prophylactic	751 (1.6)	1132 (2.2)	<0.001	1884 (1.9)
Tachycardia	866 (1.9)	876 (1.7)	0.031	1743 (1.8)
Other	215 (0.5)	267 (0.5)	0.260	483 (0.5)
Unknown/uncoded/unspecified	6195 (13.6)	7293 (14.3)	0.002	13,530 (14.0)
Aetiology, *n* (%):				
Conduction tissue disease	5219 (11.4)	5401 (10.6)	n.c.	10,623 (11.0)
Ischaemic	2100 (4.6)	2718 (5.3)	n.c.	4818 (5.0)
Therapy induced	1603 (3.5)	1978 (3.9)	n.c.	3583 (3.7)
Cardiomyopathy	1170 (2.6)	1400 (2.7)	n.c.	2572 (2.7)
Post myocardial infarction	716 (1.6)	1528 (3.0)	n.c.	2244 (2.3)
Congenital	394 (0.9)	348 (0.7)	n.c.	742 (0.8)
Other	182 (0.4)	446 (0.9)	n.c.	628 (0.6)
Unknown/uncoded/unspecified, *n* (%)	34,277 (75.1)	37,345 (73.0)	n.c.	71,690 (74.0)

^a^For 75 patients the sex is unknown, for 38 males and 22 females age is unknown

^b^ *P*-value was not calculated for aetiology because of large proportion of missing data (*n*.c.)

^c^For 1995–2005

^d^ *EPS* electrophysiological study

**Fig. 1:**
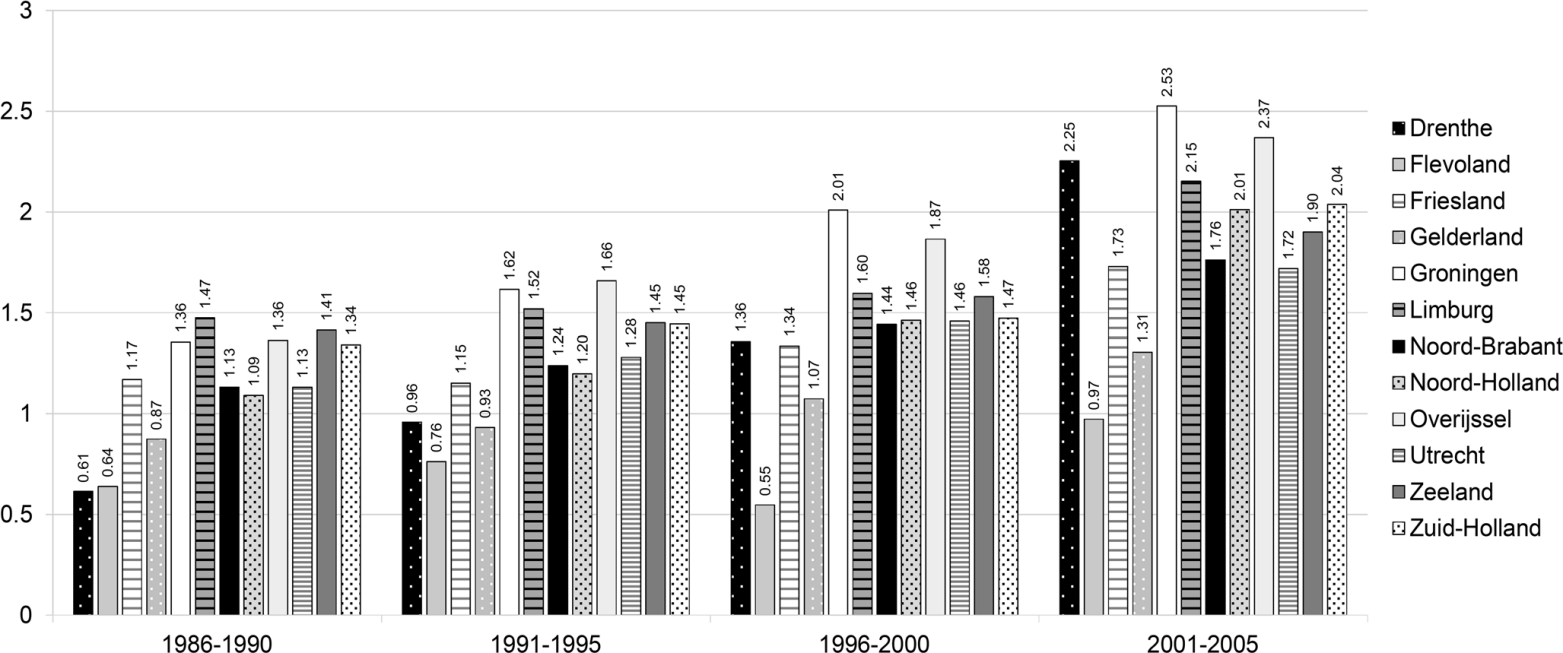
Number of first implantations per 1000 inhabitants per province. (1984 and 1985 excluded to create equal time periods, additionally no population data are available for these years for Flevoland)

One hospital performed nearly 4000 first implantations during the study period, which is approximately 4 % of the total number of first implantations. One hospital implanted more than 2600 first pacemakers (2.7 %), 5 hospitals implanted between 1900 and 2500 pacemakers each (2–2.5 %), another 36 hospitals implanted between 900 and 1900 pacemakers each (1–2 %). All other hospitals each implanted less than 1 % of the total number of first pacemakers. Eight of these hospitals did not participate in the registration until the early 1990s. Six hospitals did not start until the late 1990s or early 2000s. Two hospitals implanted less than 10 first pacemakers per year. Most pacemakers per 1000 inhabitants were implanted in the province of Groningen, followed by the provinces of Overijssel and Limburg (Fig. 1 and Table 2).

**Table 2:** Number of implants per 1000 inhabitants and distribution of population >60 years over provinces

	1986–1990^a^		1991–1995		1996–2000		2001–2005	
	Number of implants per 1000 inhabitants per province (95 % CI)	% of population >60 years^b^	Number of implants per 1000 inhabitants per province (95 % CI)	% of population >60 years	Number of implants per 1000 inhabitants per province (95 % CI)	% of population >60 years^b^	Number of implants per 1000 inhabitants per province (95 % CI)	% of population >60 years
Drenthe	0.61 (0.54–0.69)	3.1	0.96 (0.87–1.05)	3.2	1.36 (1.25–1.47)	3.3	2.25 (2.12–2.38)	3.4
Flevoland	0.64 (0.54–0.76)	0.9	0.76 (0.65–0.87)	1.1	0.55 (0.47–0.63)	1.3	0.97 (0.87–1.07)	1.4
Friesland	1.17 (1.09–1.26)	4.3	1.15 (1.06–1.24)	4.2	1.34 (1.25–1.43)	4.1	1.73 (1.63–1.83)	4.2
Gelderland	0.87 (0.83–0.92)	11.9	0.93 (0.89–0.97)	12.0	1.07 (1.02–1.12)	12.1	1.31 (1.26–1.36)	12.2
Groningen	1.36 (1.26–1.46)	4.1	1.62 (1.51–1.73)	3.9	2.01 (1.89–2.13)	3.8	2.53 (2.40–2.66)	3.7
Limburg	1.47 (1.40–1.55)	7.3	1.52 (1.45–1.59)	7.5	1.60 (1.53–1.67)	7.8	2.15 (2.07–2.24)	7.9
Noord-Brabant	1.13 (1.09–1.13)	12.8	1.24 (1.19–1.29)	13.4	1.44 (1.39–1.49)	14.2	1.76 (1.71–1.81)	14.7
Noord-Holland	1.09 (1.05–1.13)	16.7	1.20 (1.16–1.24)	16.2	1.46 (1.41–1.51)	15.7	2.01 (1.96–2.06)	15.5
Overijssel	1.36 (1.29–1.44)	6.8	1.66 (1.58–1.74)	6.8	1.87 (1.79–1.95)	6.8	2.37 (2.28–2.46)	6.8
Utrecht	1.13 (1.07–1.20)	6.3	1.28 (1.21–1.35)	6.4	1.46 (1.39–1.53)	6.4	1.72 (1.64–1.80)	6.4
Zeeland	1.41 (1.30–1.54)	2.8	1.45 (1.33–1.57)	2.8	1.58 (1.45–1.71)	2.8	1.90 (1.76–2.04)	2.8
Zuid-Holland	1.34 (1.30–1.38)	22.9	1.45 (1.41–1.49)	22.3	1.47 (1.43–1.51)	21.7	2.04 (1.99–2.09)	21.0

^a^1984 and 1985 excluded to create equal time periods, additionally no population data are available for these years for Flevoland

^b^Calculated over the years 1988–1990, no population data available for 1986 and 1987

**Fig. 2:**
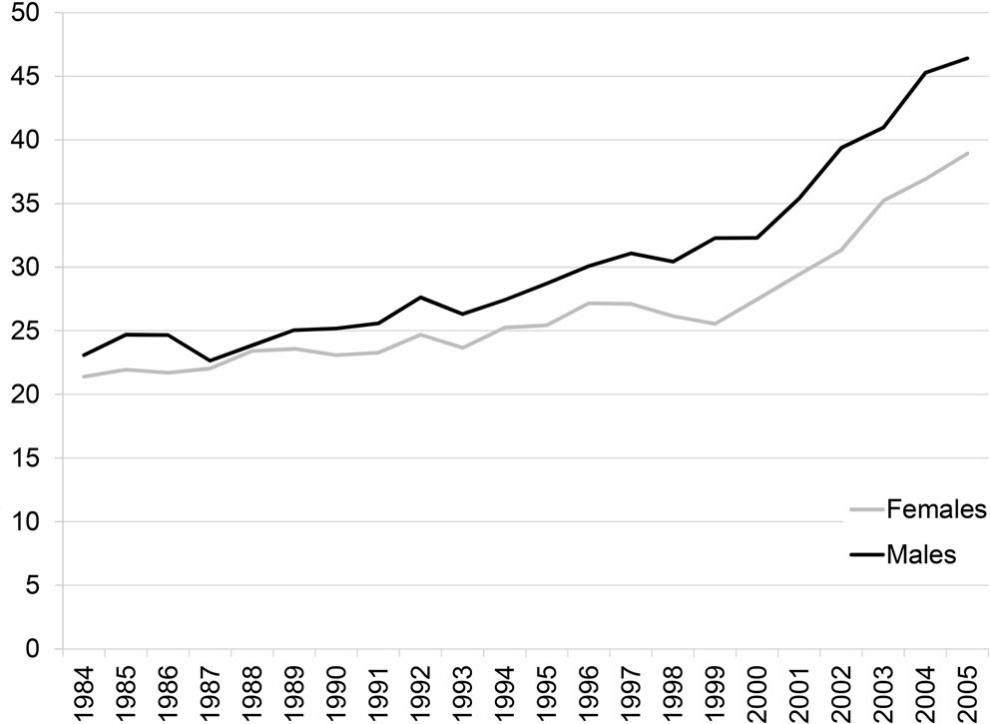
Number of first implantations per 100,000 inhabitants by sex

**Fig. 3:**
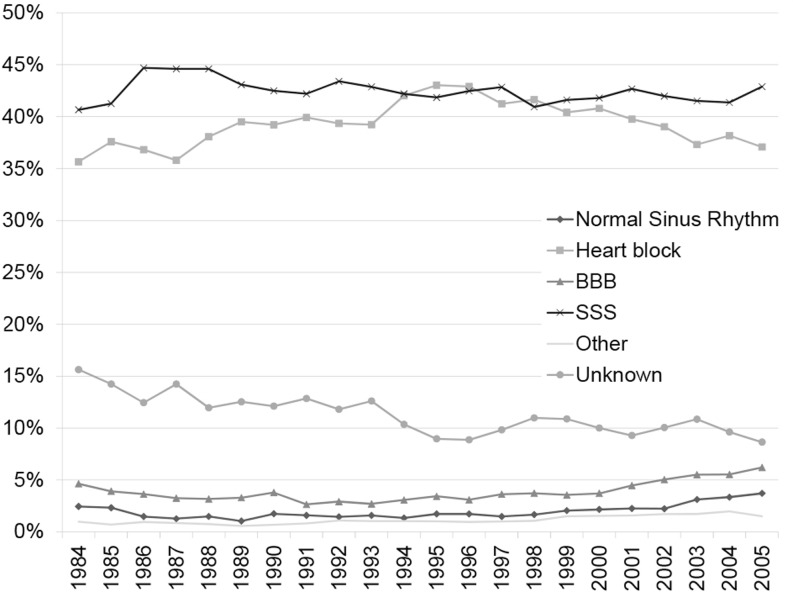
Indication for first pacemaker implantation, adjusted for number of implantations per year. (*BBB* bundle branch block; *SSS* sick sinus syndrome; normal sinus rhythm with or without abnormal electrophysiological study)

The cohort comprised 52.8 % men (*n* = 51,164). Starting in 1995 the number of implantations in men was significantly higher than in women (*p* < 0.001) in each following year. In the period before 1995 the number of implants in men was only significantly higher in the years 1985, 1986 and 1992 (Table 1, Fig. 2). Mean age for the total cohort was 72.9 years (SD 13.0). The mean age at first implantation increased from 71.1 in 1984 to 72.3 in 2005 for men (mean difference 1.2 years, *p* = 0.003) and from 72.8 to 75.1 for women (mean difference 2.3 years, *p* < 0.001). The majority of the patients were over 60 years of age (87.9 %) and pacemakers were most often implanted in the age group of 60 to 80 year olds. The percentage of first implantations in octogenarians and nonagenarians is constant over the years. However, first implantations in these groups have been increasing since 2002. The most common indications were sick sinus syndrome (*n* = 41,026; 42.3 %) and heart block (*n* = 37,682; 38.9 %). This did not change over the years (Fig. 3). The number of pacemaker implantations for both sick sinus syndrome and bundle branch block significantly differed between men and women (*p* < 0.001). All baseline characteristics are provided in Table 2.

**Fig. 4:**
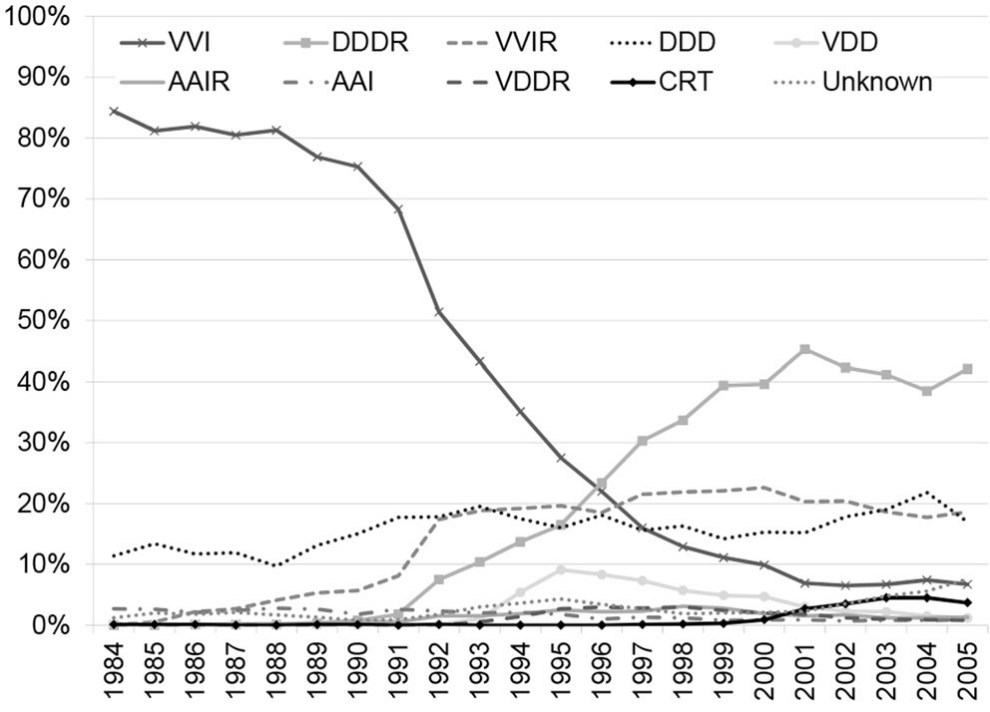
Type of pacemaker at first implantation

Pacemakers of 18 different manufacturers were implanted between 1984 and 2005. The ventricular pacing and sensing (VVI) and dual-chamber pacing and sensing, rate response (DDDR) types were the most commonly used pacemakers: 34.3 % and 23.1 %, respectively (Table 3). The other types, ventricular pacing and sensing, rate response (VVIR, 15.2 %) and dual-chamber pacing and sensing (DDD, 16.1 %), were used less often. However, during the early 1990s implantation of pacemaker types changed markedly from a mainly VVI type (single chamber systems) to a DDDR type (dual chamber systems) as depicted in Fig. 4. At least 138,225 leads were implanted with the 96,900 first pacemakers. In two thirds of the cases the leads were placed in the ventricle. For 1024 implantations (1 %) no leads were registered. For 83 % of these implantations the type of pacemaker was also not registered. More than 80 % of these pacemakers were implanted during the last 5 years of the study period.

**Table 3:** Type of pacemakers implanted in the period 1984–2005 in the Netherlands

	First pacemaker *N* = 96,900
Pacemaker type	*N* (%)
VVI	33,241 (34.3)
VVIR	14,704 (15.2)
DDD	15,636 (16.1)
DDDR	22,424 (23.1)
AAI	1500 (1.4)
AAIR	1350 (1.4)
VDD	2677 (2.8)
VDDR	1123 (1.2)
Biventricular pacing	1269 (1.3)
Unknown	2,976 (3.1)

Additionally, after the introduction of cardiac resynchronisation therapy (CRT) around 1995 [13], the use of biventricular pacing increased between 2000 and 2005. This change of pacemaker type is also reflected by the number of first implantations with three leads (*n* = 1269), 914 (72.3 %) of which for the indication dyspnoea/heart failure and 65 (5.1 %) for bradycardia. The other first implantations with three leads were for various other indications (*n* = 96, 7.6 %) and unknown, uncoded or unspecified indications (*n* = 190, 15.0 %).

## Discussion

In the Netherlands the SPRN registry was operational for more than 20 years to collect pacemaker implantations with nearly complete nationwide coverage; almost 97,000 patients received a pacemaker for the first time between 1984 and 2006. The registry showed that the number of implanted pacemakers has increased steadily over the past few decades. This increase continued in later years (2003–2012) [14].

The number of first implantations per million Dutch inhabitants was below the European average: 314, 294, and 532 implantations per million inhabitants in 2001, 2005, and 2009, respectively, compared with an average of 390, 475, and 552 implantations per million inhabitants in Europe [15–17]. Stofmeel and colleagues [18] also reported that the Netherlands had a smaller number of implantations compared with other European countries over the period 1984–1997. This may be a consequence of reluctance to use pacemakers for indications for which there was limited evidence at that time, such as asymptomatic total AV-block or asymptomatic second degree AV-block, type Wenckebach, or syncope which is not proven to be a consequence of a total AV-block in patients with a bifascicular or trifascicular block and other causes for syncope cannot be excluded. At that time there was no agreement for those indications (class 2 indications), as was formulated in the 1999 Dutch pacing guidelines [18, 19].

The difference in implantation rates between provinces does not seem to be related to differences in the age of the population. Some provinces with a higher implantation rate have a younger population than provinces with a lower implantation rate. In some provinces one or two hospitals are responsible for more than half of the implantations performed in that province. These hospitals could be ‘hot spots’ that treat patients from a wider area than the province alone. When done on a regular basis, pacemaker implantation is safe. However, operator volume appears to count when it comes to quality of care. A concentration of procedures in centres where cardiologists implant at least 50 devices per year has therefore been suggested previously [20].

Sick sinus syndrome and heart block were the major indications for pacemaker implantation. Sick sinus syndrome was significantly predominant in women. This could be attributed to the fact that sick sinus syndrome occurs more often in female than in male individuals [21]. The indications for pacemaker implantations found in our study are in line with the major indications in other European countries. During the early 1990s physiological pacing and the use of adaptive pacing frequencies with dual chamber systems (DDD(R)) were increasingly used compared with single chamber systems (VVI(R)). From the more recent World Society of Arrhythmias (WSA) surveys it appeared that indeed virtually all countries had increased percentages of DDDR pacemaker implantations [15–17, 22]. A modest increase of implantation for bundle branch block is visible over the period 2000 to 2005, especially in men. This may be explained in part by the increased use of biventricular pacemakers for cardiac resynchronisation therapy. CRT devices with ICD function (CRT-D) were not registered in this database and are therefore not included in the current analysis. Internationally, an increase of non-bradyarrhythmic indications for cardiac pacing was projected, however remained a minor indication with approximately 5 % or less in most countries [15–17, 22]. In the Netherlands this percentage remained less than 5 % for a long time, but started increasing in the early 2000s.

### Limitations

An important limitation of the data is that registration lies in many hands, which facilitates registration errors such as typographical errors and duplicate registrations of patients transferred to another hospital. We removed these duplicates whenever possible. However, some may have been overlooked due to unavailability of highly detailed patient-related data needed to distinguish duplicate records. Nevertheless, 99 % of all implantations are registered [12, 18] and the data provide useful information; therefore, errors are expected to be random with regard to indication and pacemaker properties. In contrast to the ECG data and symptoms, information on aetiology is absent in most cases. However, in cardiology practice it appears to be very difficult to establish the precise cause of cardiac or non-cardiac disease that elicits rhythm or conduction disturbances. Additionally, the type of pacemaker was registered at baseline, but the actual setting could have been changed after implantation or during follow-up.

## Conclusion

Maintaining a registry for implantable devices can serve many purposes. It provides insight into the patient population, trends regarding devices used and perhaps even more important: tracking and tracing of products in case of failures and recalls. Over the years the SPRN registry has provided input for several of these purposes, the most important being the tracking and tracing of patients in case of recalled devices. As some risks of implantable devices will only become apparent during the actual clinical use after marketing of the device, registries can serve a purpose in identifying those as well.

In conclusion, analysis of the SPRN database has shown that the frequency of first implantation of pacemakers has steadily increased in the Netherlands and that trends in indications for implantation and pacemaker type are in line with other European countries.
